# Work-Time Control and Exhaustion: Internal Work-to-Home Interference and Internal Home-to-Work Interference as Mediators

**DOI:** 10.3390/ijerph19063487

**Published:** 2022-03-15

**Authors:** Laura Vieten, Anne Marit Wöhrmann, Alexandra Michel

**Affiliations:** 1 Federal Institute for Occupational Safety and Health (BAuA), Friedrich-Henkel-Weg 1-25, 44149 Dortmund, Germany; woehrmann.annemarit@baua.bund.de (A.M.W.); michel.alexandra@baua.bund.de (A.M.); 2 Department of Psychology, Heidelberg University, Hauptstraße 47-51, 69117 Heidelberg, Germany

**Keywords:** autonomy, flexible working hours, Germany, national survey, occupational health, work–family conflict

## Abstract

Strong work-time control (WTC) has been linked to reduced employee exhaustion, with work-to-home interference as an underlying mechanism. In this study, we aimed to investigate the mediation effect of both directions of internal work–home interference, namely internal work-to-home interference (IWHI) and internal home-to-work interference (IHWI). The analysis is based on data from the 2015, 2017, and 2019 BAuA-Working Time Survey, a representative German panel study. Cross-lagged panel models were estimated separately for IWHI and IHWI, based on the balanced panel (*n* = 3390). We investigated the hypothesized indirect as well as potential direct, reversed, and reciprocal effects of the constructs. WTC had a small but significant indirect effect on exhaustion via IWHI. Contrary to assumptions, WTC positively affected IHWI. Unexpectedly, there was no significant effect of IHWI on exhaustion. Hence, only IWHI was identified to mediate WTC’s effect on exhaustion. This implies that WTC helps employees avoid exhaustion from psychological preoccupation with work during free time. In addition, analyses suggested reversed and reciprocal relationships between the investigated constructs. Further investigation is needed to explore the role of psychological preoccupation with private matters during work time in the context of WTC and employee well-being.

## 1. Introduction

Irregular working hours such as night shifts, a variable number of daily or weekly working hours, or on-call work are associated with several adverse mental health outcomes, including sleep problems, depressed mood, or anxiety [1,2,3]. As irregular working hours are unavoidable in some sectors, for example, in health care, protection against the adverse effects is crucial. One way to protect employees against the negative consequences of irregular working hours is providing them with control over working hours. For instance, Tucker et al. [4] found that work-time control (WTC) reduces the negative impact of frequent night working on sleep disturbances. Bohle et al. [5] noticed a reduced dissatisfaction with working hours, related to work–home conflict and mental health, for call center workers with working hour variability when WTC was high.

WTC can be defined as “employee’s possibilities to control the duration, position, and distribution of his or her worktime, that is, autonomy with regard to worktime” [6,7] (p. 503). This autonomy can be expressed in multiple forms, such as control over beginning and ending the workday (flextime), when to take work breaks, or when to take days off or vacation when needed [8,9]. WTC is common in the European Union. Already in 2015, 44 percent of EU employees had some control over their working hours [10]. The COVID-19 pandemic has presumably increased this percentage, not least because of the associated increase in remote work.

In line with the job demands–resources theory [11], WTC can be an essential job resource preventing exhaustion. The job demands–resources theory [11] proposes that job resources can buffer adverse effects of job demands such as irregular working hours on employees’ job strain. A consequence of intensive strain is exhaustion [12], which is a pivotal indicator of employee well-being and linked to several mental health outcomes such as depression [13] or anxiety [14]. Indeed, previous studies found diminished exhaustion for employees with high WTC [15,16,17]. For instance, in a study by Kattenbach et al. [15], which used data from employees of 17 human services organizations in Germany, WTC—operationalized as the possibility to vary working time—was related to lower exhaustion. Another study with a large sample of Japanese daytime and shift workers revealed associations between high WTC and fewer sleep problems and fatigue [18]. 

Research into why or how WTC relates to employee exhaustion has suggested that work-to-home interference is a potential mediator [16,17]. However, work–home interference, defined as a conflict resulting from somewhat incompatible work and home demands [19], is bidirectional: work and home domains can interfere with one another, although researchers have focused on work-to-home rather than home-to-work interference [16,17]. Thus, we examined both directions of work–home interference as mediators. Moreover, we focused on *internal* work–home interference as a specific subdimension that captures “psychological preoccupation with one domain of life (e.g., work) while within the role boundaries of another domain of life (e.g., family)” [20] (p. 518). It includes thinking or even ruminating about work issues at home and vice versa. In contrast to general work–home interference, this subdimension focuses on employees’ mental processes rather than their behavior. Due to this psychological preoccupation, their ability to engage in work or home roles is restricted. 

Consequently, we investigated two possible mediators for the effect of WTC on exhaustion: internal work-to-home interference (IWHI) and internal home-to-work interference (IHWI). To elaborate on IWHI and IHWI as possible mediators in the WTC-exhaustion relationship, we first outlined our assumptions about how WTC relates to IWHI/IHWI and then about how IWHI/IHWI relate to exhaustion. Then we situated our study within previous research investigating (internal) work–home interference as a mediator in the relationship between WTC and employee mental health, including exhaustion.

### 1.1. WTC and IWHI/IHWI

Kossek and Lautsch’s [21] cross-level model of work–family boundary management styles states that individuals’ control over their boundary management styles reduces work–home interference because they feel able to manage interactions between work and home. Following this theoretical assumption, we argue that WTC allows employees to manage boundaries between work and home as they desire [21], which then prevents work and home responsibilities from conflicting [8,22] and keeps domains from intruding into one another. Therefore, we assume that WTC helps employees to avoid thinking about work during nonwork time and vice versa. For example, imagine a scenario in which an employee must seek car repairs and find a time that aligns with repair shop hours. With WTC, the employee can adjust work hours to take the car to the shop and then focus on work, reducing thoughts about the car during work and thus IHWI. In another scenario, an employee is deeply engaged in a pressing project. With WTC, the employee can choose to continue and finish working on the task rather than postponing it to another day, reducing thoughts about the task during free time and thus IWHI. In line with these scenarios, relations between unfinished and interrupted tasks and rumination have already been found in research [23,24]. For instance, Syrek and Antoni [24] found that employees’ feelings of not having finished the week’s tasks enhance their rumination about work-related problems during the weekend. Thus, based on these considerations, we assume that WTC reduces IWHI/IHWI.

### 1.2. IWHI/IHWI and Exhaustion

Both IWHI and IHWI involve issues from one domain intruding into the other, causing employees to ruminate on issues at the wrong times [20]. Thus, following the model of prolonged stress-related activation [25], we argue IWHI and IHWI to entail sustained cognitive activation leading to prolonged physiological stress responses. This can result in drained energy resources, causing exaggerated exhaustion in the long term. Several studies supporting this assumption found that a lack of psychological detachment from work, a construct similar to IWHI [26], relates to employees’ exhaustion [27]. Thus, based on these considerations, we assumed that IWHI/IHWI leads to exhaustion.

### 1.3. IWHI/IHWI as a Mediator

Research on the relationship between WTC and exhaustion has suggested that general work-to-home interference is a potential mediator [17]. For instance, Yu [17] found that work-to-home interference mediated the negative relationship between WTC and emotional exhaustion in a sample of employees from a U.S. healthcare company. Moreover, using data from a large Swedish study, Albrecht et al. [22] found work-to-home interference to mediate the relationship between WTC and mental health, specifically depressive symptoms.

However, research specifically focusing on internal work–home interference has been relatively scarce. To our knowledge, both IWHI and IHWI have mostly been ignored in previous research as possibly mediating the relationship between WTC and exhaustion or other aspects of employee mental health. An exemption is a study by Moen et al. [16], who investigated whether participation in a corporate initiative focused on attending to work results instead of time spent working improved employee health. They found an indirect effect in which participation in the initiative increased WTC, which decreased IWHI, operationalized as negative work-to-home spillover. IWHI, in turn, diminished emotional exhaustion. In other words, they found that IWHI mediated the relationship between WTC (caused by participation in the initiative) and exhaustion.

**Figure 1:**
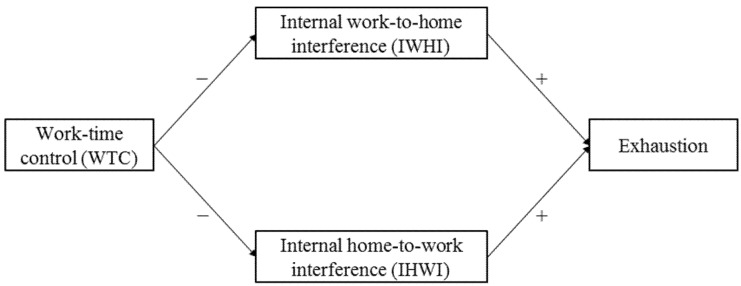
Hypothesized relationships between work-time control (WTC), internal work-to-home interference (IWHI), internal home-to-work interference (IHWI), and exhaustion. − = negative effect; + = positive effect.

Overall, based on the theoretical assumptions made and previous empirical findings, we hypothesized IWHI and IHWI to mediate the effect of WTC on employees’ exhaustion as shown in Figure 1. More specifically, we hypothesized that:

**Hypothesis** **1.** *WTC reduces employee exhaustion, with IWHI as mediator.*

**Hypothesis** **2.** *WTC reduces employee exhaustion, with IHWI as mediator.*

We investigated these hypotheses in an empirical study that builds on the studies of Moen et al. [16] and Albrecht et al. [22] by combining part of Moen et al.’s research questions with the cross-lagged panel design of Albrecht et al. Thereby, our study has several methodological strengths. First, we use a large German sample including employees from various branches, occupations, and organizations. Second, we use panel data from three measurement points allowing a fully longitudinal design. Moreover, we considerably extend existing research and enhance the understanding of how WTC relates to employee exhaustion by examining both directions of internal work–home interference as underlying mechanisms. Because the constructs could relate in various directions [17,22], we include the investigation of direct, reversed, and reciprocal effects. 

## 2. Materials and Methods

### 2.1. Data Collection

We used data from the BAuA-Working Time Survey, a representative biennial panel study collected in 2015 (T1), 2017 (T2), and 2019 (T3). The aim of this survey is to collect longitudinal data on working (time) conditions, health, and well-being for a part of the German workforce. Professional interviewers used computer-assisted telephone interviews to collect data from individuals 15 years or older in paid employment for at least 10 h per week. Further information on the survey is available elsewhere [28,29,30,31].

### 2.2. Sample

We restricted the sample to dependent employees 65 years or younger. To ensure a balanced panel, we included only participants who took part in all three waves of data collection. Thus, our final sample included 3390 employees, almost half of whom were women (48%). The average age was 47.35 years (SD = 8.60). About three-quarters of the participants (74%) lived with a partner and 39 percent had at least one underage child in the household. The majority of the sample was highly educated (58%). They came from various branches and occupations and worked 39.09 (SD = 10.49) hours per week on average. Following Granderath et al. [32], we ran an additional analysis with an unbalanced panel including all dependent employees 65 years or younger, without restrictions regarding participation in all three measurements (*n* = 17,918; see Appendix A, especially Table A1). To assess differences between “stayers” who participated in all three waves and “leavers” who did not, we conducted Mann-Whitney U tests, Fisher’s exact tests, and a multiple logistic regression including study variables and control variables (Appendix A: Table A2 and Table A3).

### 2.3. Measures

All measures were assessed at T1, T2, and T3. All items were from well-validated scales and rated on five-point Likert scales. As Table 1 shows, scales had acceptable internal consistencies (Cronbach’s alphas) at all three measurement points (α ≤ 0.76).

*WTC* was measured using three slightly adapted items from the German translation [33] of the control-over-work time scale by Valcour [9]. For example, “How much control do you have over when you begin and end each workday?” was used. We also used a question adapted from the German version of the Copenhagen Psychosocial Questionnaire [34]: “How much control do you have over when you take a break?” Thus, we used four items to measure WTC, with responses on a five-point Likert scale from 1 (very little control) to 5 (very high control). Confirmatory factor analyses (CFAs) results varied between measurements (χ²(2) = 63.817–100.356, *p* < 0.001; CFI = 0.974–0.984; RMSEA = 0.095–0.120), while exploratory factor analyses (EFAs) consistently revealed one factor with an eigenvalue above 1.

*IWHI* and *IHWI* were each assessed using a translated and adapted subscale of Carlson and Frone’s [20] work–family interference scale. Both subscales included three items, rated on a five-point Likert scale from 1 (does not apply at all) to 5 (applies completely). Example items include, “When I am at home, I often think about things I need to accomplish at work” (IWHI) and “When I am at work, I often think about home-related problems” (IHWI). Because models with three items are fully identified, we could not use CFAs to test assumptions that each of the three items represents one dimension. EFAs revealed a one-factor solution (eigenvalue above 1) for both constructs at all three measurements. In addition, we performed CFAs to justify our examination of IWHI and IHWI as distinct constructs. A two-factor model (χ²(8) = 22.567–26.361, *p* < 0.001; CFI = 0.998; RMSEA = 0.023–0.026), fitted the data better than a one-factor model (χ²(9) = 3359.096–4024.779, *p* < 0.001; CFI = 0.509–0.587; RMSEA = 0.331–0.363), supporting our assumption of two distinct but correlated constructs.

*Exhaustion* was measured with four items from the corresponding subscale of the Oldenburg Burnout Inventory by Demerouti et al. [12]. For example, “After work, I tend to need more time than in the past in order to relax and feel better.” The answer format was a five-point Likert scale from 1 (does not apply at all) to 5 (applies completely). CFA results differed (χ²(2) = 82.843–102.191, *p* < 0.001; CFI = 0.976–0.977; RMSEA = 0.109–0.122). EFAs showed one factor with an eigenvalue above 1 at all three measurements.

Aligned with research regarding WTC, work–home interference, and/or exhaustion [16,22,35], we used sociodemographic aspects and working conditions as *control variables*. Specifically, variables included gender, age, education level, living with a partner, underage child in household, weekly working hours, regular day work, and requirement levels according to the German classification of occupations (KldB 2010) [36]. The need to control for some of these variables also resulted from their association with dropout (see Table A2 and Table A3 in Appendix A).

### 2.4. Statistical Analysis

We applied structural equation modeling (path analysis) to test associations between WTC, IWHI, IHWI, and exhaustion. We used cross-lagged panel models to control for stability and cross-sectional relationships. We analyzed IWHI and IHWI separately to reduce model complexity. To contend with non-normally distributed variables, we used maximum likelihood estimation with robust standard errors (MLR) and full information maximum likelihood (FIML) to account for missing data. We examined indirect effects using bias-corrected bootstrapping with 5000 draws [37]. MLR cannot be applied with bootstrapping, so we used the maximum likelihood estimator instead. Data preparation, including screening for normality, outliers, multicollinearity, and descriptive statistics, was executed with SPSS version 26. Cross-lagged panel analyses were performed with Mplus version 8 [38].

Following Albrecht et al. [22], we tested for longitudinal mediation using a step-wise analytic approach adapted from Cole and Maxwell [39] and Little [40]. By estimating and comparing a series of competing models, we could test the relevance and robustness of the assumed mediation paths and the direct, reversed, and reciprocal effects. Lagged path estimates were constrained to be equal over time in all models. For example, we fixed WTC T1→IWHI T2 to the same coefficient as WTC T2→IWHI T3. Specifically, we compared (a) a stability model (M0) including cross-sectional correlations between constructs and first-order (e.g., WTC T1→WTC T2) and second-order (e.g., WTC T1→WTC T3) auto-regressive paths; (b) a causal mediation model (M1) that additionally contained cross-lagged paths of assumed mediation, that is, paths from WTC to IWHI/IHWI and from IWHI/IHWI to exhaustion; (c) a reversed mediation model (M2) with additional paths from IWHI/IHWI to WTC and from exhaustion to IWHI/IHWI; (d) a direct effect model (M3) additionally including direct paths from WTC to exhaustion; (e) a reversed direct effect model (M4) that additionally contained paths from exhaustion to WTC; and (f) a final model (M5) including the tested paths that had improved model fit (e.g., assumed causal mediation, reversed mediation, direct and reversed direct effects), and then control variables, with nonsignificant paths removed. We regressed control variables on all study variables. Gender, age, and education level were assumed to be time-stable and therefore regressed on the constructs at T1 only. If mediation paths remained significant in M5, we calculated indirect effects. 

We assessed model fit by root mean square error of approximation (RMSEA) [41] and comparative fit index (CFI) [42]. Acceptable fit is indicated by RMSEA values of <0.080 and CFI values of >0.900 [40]. We also reported chi² values (χ2) but did not use them to evaluate model fit because they can be affected by large samples and thus may reject appropriate models. To compare nested models, we used Bayesian information criterion (BIC) [43] and the Satorra-Bentler chi² difference test [44]. Given the large sample, we set the significance level to 0.01 for all tests. 

## 3. Results

### 3.1. Descriptives

Table 1 shows descriptive statistics, internal consistencies, and correlations between study variables across all three measurement points. As expected, WTC was significantly and negatively correlated with IWHI and exhaustion across all measurement points. Surprisingly, WTC was significantly and positively correlated with IHWI. In addition, both IWHI and IHWI were significantly positively related to exhaustion, with larger coefficients for IWHI and exhaustion than for IHWI and exhaustion (0.24–0.35 vs. 0.05–0.08).

### 3.2. IWHI as Mediator (Hypothesis 1)

**Figure 2:**
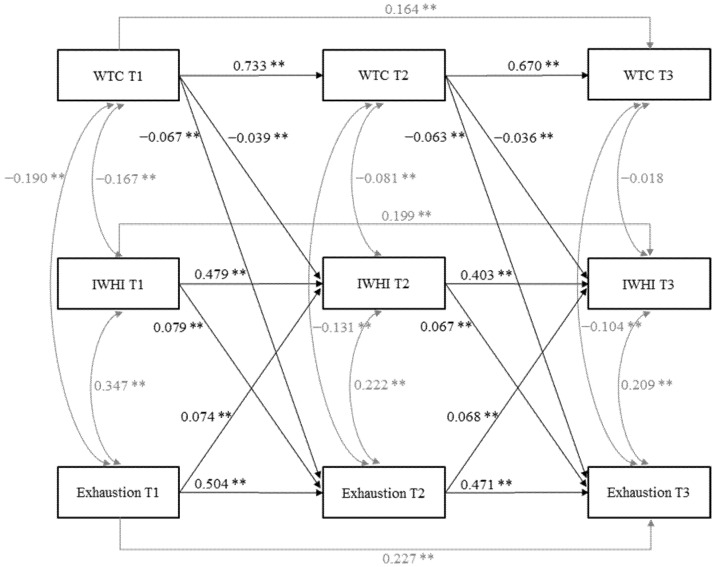
Final model (M5) with standardized coefficients for WTC, IWHI, and exhaustion. For clarity, control variables (gender, age, education level, living with a partner, underage child in household, weekly working hours, regular day work, and requirement level) are not shown. * *p* < 0.01. ** *p* < 0.001.

Model fit was improved by adding causal mediation pathways via IWHI (M1: WTC→IWHI β = −0.027, *p* < 0.01; IWHI→exhaustion β = 0.071, *p* < 0.001) to a model that allowed only cross-sectional correlations and auto-regressive paths (M0), as indicated by model fit indexes and chi² difference tests (Table 2). Model fit was also improved by entering reversed mediation pathways (M2: exhaustion→IWHI β = 0.085, *p* < 0.001; IWHI→WTC β = −0.012, *p* = 0.131) and direct effects (M3: WTC→exhaustion β = −0.060, *p* < 0.001). However, as shown in Table 2, adding reversed direct effects (M4: exhaustion→WTC β = −0.021, *p* = 0.032) did not substantially improve model fit. Thus, our final model (M5) included causal and reversed mediation paths and direct effect paths. However, we pruned the paths from IWHI to WTC as part of the reversed mediation paths because they were nonsignificant before and after we included control variables. Including control variables modified estimates only slightly. All paths of causal mediation remained significant (WTC→IWHI β = −0.035, *p* < 0.001, IWHI→exhaustion β = 0.063, *p* < 0.001; Figure 2). The indirect effect from WTC to exhaustion via IWHI was −0.002 (SE 0.001, 95% CI −0.004–−0.001, *p* < 0.01; standardized −0.003, SE 0.001). Thus, we found support for Hypothesis 1, assuming IWHI as a mediator in the relationship between WTC and exhaustion. 

Results based on the unbalanced panel supported results based on the balanced panel. All paths in the final model also reached significance. Standardized estimates slightly differed in magnitude. However, the final model based on the unbalanced panel had one more path that is significant: the reversed direct effect paths from exhaustion to WTC.

### 3.3. IHWI as Mediator (Hypothesis 2)

**Figure 3:**
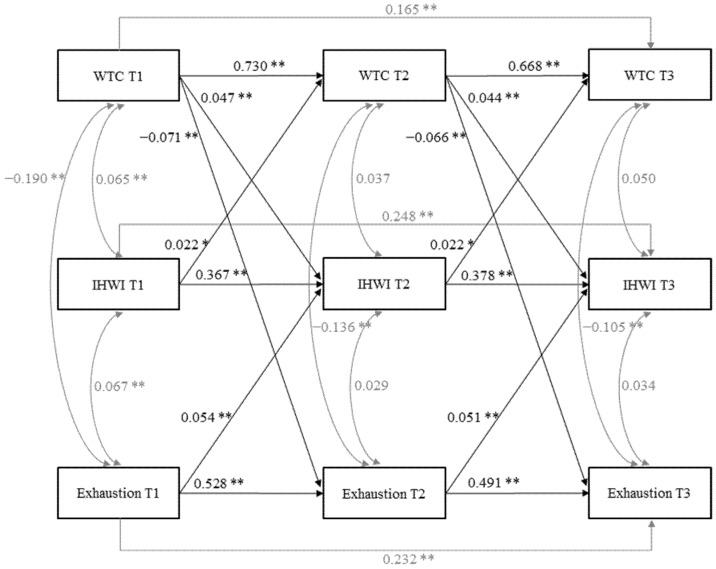
Final model (M5) with standardized coefficients for WTC, IHWI, and exhaustion. For clarity, control variables (gender, age, education level, living with a partner, underage child in household, weekly working hours, regular day work, and requirement level) are not shown. * *p* < 0.01. ** *p* < 0.001.

Model fit was improved by entering causal mediation pathways via IHWI (M1: WTC→IHWI β = 0.022, *p* < 0.01; IHWI→exhaustion β = 0.021, *p* = 0.060) to the null model (M0; see chi² difference test in Table 2, however, results regarding fit indexes varied). We also found a significantly better fit when we included reversed mediation pathways (M2: exhaustion→IHWI β = 0.045, *p* < 0.001; IHWI→WTC β = 0.022, *p* = 0.036) and direct effects (M3: WTC→exhaustion β = −0.064, *p* < 0.001). Fit indexes differed for reversed direct effects (M4: exhaustion→WTC β = −0.025, *p* < 0.01), while chi² difference test again indicated a significantly better fit. Thus, our final model (M5) included causal mediation paths, reversed mediation paths, direct effect paths, and reversed direct effects. However, we pruned the paths from IHWI to exhaustion because they were nonsignificant in the final model, before and after we included control variables. In addition, we pruned paths from exhaustion to WTC because they became nonsignificant when we included control variables. Paths from IHWI to WTC were nonsignificant before we included control variables, but we kept them in the final model because they were significant after we included control variables (Figure 3). Calculating the indirect effect from WTC to exhaustion via IHWI was impossible because we had pruned the nonsignificant paths from IHWI to exhaustion. Thus, we failed to find support for Hypothesis 2 regarding IHWI as a mediator.

Results based on the unbalanced panel differed from results based on the balanced panel. The final model based on the unbalanced panel had two more paths that are significant: from IWHI to exhaustion and the reversed direct effects paths from exhaustion to WTC. The reversed paths from IHWI to WTC were significant in the balanced panel, but insignificant in the final model based on the unbalanced panel.

## 4. Discussion

In this study, we analyzed panel data from a large German study to investigate if both IWHI and IHWI mediate the relationship between WTC and exhaustion. The data support Hypothesis 1, expecting WTC to reduce exhaustion via reduced IWHI. Thus, they support the contentions that WTC is a critical job resource that reduces employees’ IWHI, that is, it allows employees to stop rumination processes and detach psychologically from work during free time, thereby reducing exhaustion. However, the data fail to support Hypothesis 2, in which we assumed IHWI to mediate the relationship between WTC and exhaustion. 

Drawing on considerations of Kossek and Lautsch’s [21] cross-level model of work–family boundary management styles and previous research on unfinished and interrupted tasks [23,24] and rumination, we assumed and find WTC to reduce IWHI. Thus, our results support this model’s assumption that WTC or its associated control over boundary management can reduce work–home interference. They additionally extend this model by showing that this assumption also holds in the specific context of the subdimension of internal work-to-home interference. However, contrary to expectations, we found a positive effect of WTC on IHWI. Thus, our results indicate that WTC does not prevent employees from thinking about private matters at work. An explanation could be that employees used WTC to form flexible and permeable work–home boundaries, allowing the home domain to spill over to the work domain. Few researchers have investigated the relationship between WTC and IHWI, although Moen et al. [45] found contrary results indicating no significant relation. Hence, future research is needed to investigate the relationship between WTC and IHWI. Besides, we recommend future investigation of moderating variables of this relationship to determine how to attenuate a potentially positive relationship.

Furthermore, we found IWHI to lead to exhaustion. This is in line with the model of prolonged stress-related activation [25] and research showing a relation between lack of detachment and exhaustion [27]. In contrast, IHWI does not significantly affect exhaustion. Nevertheless, bivariate correlations are consistently significantly positive, albeit small, and analyses based on the unbalanced panel indicate that IHWI is positively related to exhaustion. Thus, future studies need to consider that we cannot unambiguously establish whether increased thoughts about private issues during work are associated with exhaustion. Regarding general home-to-work interference, a previous meta-analysis of panel studies already showed that it predicts job-related strain, including exhaustion [46], but that the effects are weaker than those of work-to-home interference are. 

Overall, the results suggest that IWHI but not IHWI mediates WTC’s effect on exhaustion. As far as we know, research has not examined whether IHWI mediates the relationship between WTC and exhaustion or other aspects of employee health. However, our results for IWHI align with Moen et al. [16], who used data collected at two measurement points to indicate that IWHI mediates the relationship between WTC and exhaustion. Moreover, our results correspond with studies showing that general work-to-home interference mediates WTC’s relationship with exhaustion [17] and depressive symptoms [22]. Similar to our study, Albrecht et al. [22] found a significant but small indirect effect of WTC on depressive symptoms via work-to-home interference when using a cross-lagged panel model with two-year intervals. They explained the small size of WTC’s effect by the fact that WTC is a specific part of a psychosocial work environment. This explanation also applies to our study. Overall, IWHI appears to have small but stable mediation effects. 

Aligned with earlier research [15,17], we found that WTC directly affects exhaustion. This result fits the job demands–resources theory [11] stating that job resources protect against exhaustion. However, it also extends the assumption of this theory, because the job demands–resources theory assumes only a buffer effect and not a direct effect of job resources on employee strain and exhaustion. Our findings are in line with previous research showing that job resources such as social support are directly related to exhaustion [47]. In principle, we assume that this direct effect is caused by other mediating variables not investigated in this study. These may include other subdimensions of work–home interference, such as external work–home interference, or sleep problems, as Tucker et al. [48] found in associating WTC with accidents. 

Furthermore, we found evidence for reversed and reciprocal effects. Exhaustion had reversed effects on IWHI and IHWI, indicating that exhausted employees may lack sufficient energy resources to manage demands from both work and home, resulting in higher levels of IWHI and IHWI. We are unaware of any study finding a reversed or reciprocal relationship of IWHI or IHWI with exhaustion, but in some studies researchers have found reciprocal relationships of general work-to-home and home-to-work interference with exhaustion [17,46]. Our results support and extend those findings by showing that the reverse effect of exhaustion on work–home interference holds for internal work–home interference.

Moreover, after including control variables, IHWI has a reverse effect on WTC, possibly because IHWI changes perceptions of WTC or because IHWI objectively changes the work environments, including WTC. However, the analyses based on the unbalanced panel do not find this reverse effect. Future studies should therefore investigate whether extensive IHWI indeed increases WTC. The same holds for the reverse effect of exhaustion on WTC found only in the analyses based on the unbalanced panel.

### 4.1. Strengths, Limitations, and Future Research

This study is strong in its use of data from a large sample of German workers collected at three measurement points. Using a cross-lagged panel model allowed us to control for stability and cross-sectional relationships and investigate different relationships among the examined constructs. We provide a more nuanced understanding of both directions of internal work–home interference as mediating the relationship between WTC and exhaustion. 

Nevertheless, the study is not free from limitations. A common method bias affecting self-reported data might play a role. Our cross-lagged panel models might incur bias from mixing variance between and within persons. Moreover, we cannot distinguish between two commonly differentiated subdimensions of WTC: control over daily working hours and control over taking time off. Research has already examined this two-dimensional structure [49], but EFAs consistently revealed one factor with an eigenvalue above 1 for the four items used, so we decided to consider WTC as a unidimensional construct.

Furthermore, attrition and associated problems such as biased parameters and inaccurate standard errors could be problematic [50]. Analyses (Appendix A: Table A2 and Table A3) showed significant differences between stayers and leavers. We reduced biases as much as possible. We performed our analyses for both the balanced and the unbalanced panel to assess robustness. As often recommended [50,51], we used the FIML technique and included auxiliary variables as control variables in our models. More precisely, we used socio-demographic aspects and working conditions related to dropout. For our constructs, the values measured at T1 are assumed to be good estimators of missing values at T2 and T3 [51].

Another limitation is a potentially limited generalization to other countries and employee groups, such as self-employed persons, resulting from our sample of dependent employees in Germany. In Germany, for example, the Working Hours Act imposes certain regulations to protect employee health, allowing employees to only shift and extend their working hours within certain limits, even if they have a high level of WTC. Therefore, it is conceivable that for self-employed persons or countries with a different legal and sociocultural framework, distinct relationships between WTC, IWHI/IHWI, and exhaustion may emerge. For example, WTC may be more closely related to worker self-exploitation resulting in overtime work and adverse effects on work–home interference and exhaustion. Evidence for this assumption is provided by a study by Lee et al. [3] in which Korean workers with WTC had longer and more variable, i.e., irregular, weekly working hours and reported more depressive symptoms and anxiety than those without WTC. Likewise, several studies show that cultural and national aspects can influence both the job resources–strain relationship [52] and the work–home interface [53,54]. Thus, we recommend that future studies investigate the relationships between WTC, IWHI/IHWI, and exhaustion for other employment types and other countries.

Finally, our results strongly depend on the choice of a 2-year time lag between data collection waves. Although other WTC studies have also chosen 2-year time intervals [22,48], we cannot be certain that this is the “real” period, during which effects unfold. Therefore, future studies should try to identify the most optimal time lags. 

In addition to the research needs already described, further research questions remain open. In our study, we assumed and found that WTC is a crucial job resource preventing employees from exhaustion. However, other studies indicate negative associations between a high level of WTC and employee health [3,55]. Usually, this is explained by the fact that employees use their high levels of WTC to work irregular hours, such as overtime, that impede recovery and thus detrimentally affect health. For instance, in a large-scale study in Finland, a u-shaped relationship between WTC and sleep disturbances was found, but only for employees working more than 40 h per week [55]. Thus, we endorse further studies investigating under which circumstances WTC may lead to negative associations and what this means for the associations found in our study. Against the background of an increase in remote work, we suggest considering especially employees who telework. This is particularly important because remote work is associated with both a high level of WTC and a high level of boundaryless and thus often irregular working hours [56].

### 4.2. Implications for Practice

Organizations need a rested, healthy, productive workforce. Our findings indicate that by granting employees WTC, organizations can support employees in psychological detachment from work and in using their free time to recuperate and avoid exhaustion. Thus, organizations should provide their employees with a certain level of WTC. For instance, they can promote flexible working time arrangements such as flextime, allowing their employees to decide when to begin and end their work (within a legal and considerable organizational framework, of course). Additionally, they could provide employees with agency over when to take work breaks or days off.

Our results suggest that employees without regular day work (working hours usually not between 7 am and 7 pm) usually have a lower level of WTC (see Table 1). However, previous studies [4,5] indicate that WTC is a crucial resource, especially for employees with irregular working hours, because it can buffer their adverse effects on employee health. Therefore, we would like to encourage organizations to provide WTC, especially to workers with irregular work hours. Previous research has also suggested that employees with high WTC may be tempted to work irregular working hours such as overtime. Organizations should therefore support their employees in responsibly managing their WTC.

Building on our finding that WTC has a positive effect on IHWI, organizations should train employees on additional boundary management strategies, especially those preventing rumination processes. Michel et al. [57] and Althammer et al. [58] have shown that the use of mindfulness as a cognitive–emotional segmentation strategy is beneficial in this context.

Our results also indicate that exhaustion leads to more IWHI and IHWI. Therefore, we advise organizations to provide additional actions to prevent exhaustion, such as boundary management trainings [59].

## 5. Conclusions

By investigating both IWHI and IHWI as mediators, this study contributes to a more comprehensive understanding of work–home interference as mediating the relationship between WTC and exhaustion. Consistent with other research [16], we found a mediation effect of IWHI. Thus, results support the assumption of WTC as a vital job resource allowing employees to detach psychologically from work during free time and thus reducing exhaustion. Regarding IHWI, results diverge from expectations: WTC positively affects IHWI, but IHWI is not significantly related to exhaustion. However, considering the lack of previous studies and the differing results based on the unbalanced panel, future research should continue to investigate the relationships between WTC, IHWI, and exhaustion, including reverse and reciprocal effects.

## Figures and Tables

**Table 1:** Descriptive statistics, internal consistencies, and correlations among study variables (*n* = 3334–3390).

Variable ^a^	M	SD	1	2	3	4	5	6	7	8	9	10	11	12	13	14	15	16	17	18	19	20
1. Gender T1	0.48	0.50	-																			
2. Age T1	47.35	8.60	0.10 **	-																		
3. Education level T1	0.58	0.49	−0.08 **	0.02	-																	
4. Living with partner T1	0.74	0.44	−0.07 **	0.08 **	0.08 **	-																
5. Child in household T1	0.39	0.49	−0.03	−0.25 **	0.06 **	0.30 **	-															
6. Weekly working hours T1	39.09	10.49	−0.44 **	−0.01	0.17 **	−0.04	−0.09 **	-														
7. Regular day work T1	0.85	0.36	0.03	0.02	0.20 **	0.06 **	0.04	0.01	-													
8. Requirement level T1	0.56	0.50	−0.16 **	−0.01	0.58 **	0.09 **	0.09 **	0.23 **	0.22 **	-												
9. WTC T1	3.40	1.10	−0.16 **	−0.03	0.13 **	0.09 **	0.07 **	0.10 **	0.24 **	0.23 **	0.78 ^b^											
10. WTC T2	3.41	1.12	−0.15 **	−0.03	0.14 **	0.09 **	0.06 *	0.09 **	0.27 **	0.21 **	0.79 **	0.79										
11. WTC T3	3.46	1.11	−0.16 **	−0.03	0.12 **	0.09 **	0.07 **	0.11 **	0.25 **	0.20 **	0.74 **	0.79 **	0.79									
12. IWHI T1	2.53	1.10	0.01	0.06 *	0.17 **	0.03	−0.01	0.15 **	0.04	0.18 **	−0.11 **	−0.10 **	−0.10 **	0.83								
13. IWHI T2	2.42	0.99	0.01	0.04	0.10 **	−0.01	−0.02	0.12 **	0.02	0.11 **	−0.10 *	−0.12 **	−0.11 **	0.55 **	0.82							
14. IWHI T3	2.55	1.01	0.02	0.05 *	0.05 *	0.00	−0.03	0.08 **	0.03	0.07 **	−0.11 **	−0.14 **	−0.12 **	0.46 **	0.51 **	0.80						
15. IHWI T1	1.85	0.76	−0.12 **	−0.09 **	−0.10 **	−0.04	0.04	0.08 **	−0.04	−0.10 **	0.05 *	0.05 *	0.05 *	0.05 *	0.05 *	0.06 **	0.76					
16. IHWI T2	2.03	0.81	−0.11 **	−0.12 **	0.11 **	−0.03	0.03	0.03	−0.03	−0.11 **	0.05 *	0.06 **	0.07 **	−0.01	0.10 **	0.03	0.42 **	0.78				
17. IHWI T3	2.00	0.80	−0.10 **	−0.08 **	−0.10 **	−0.02	−0.01	0.05 *	−0.02	−0.09 **	0.06 **	0.05 *	0.08 **	0.02	0.06 **	0.15 **	0.42 **	0.46 **	0.79			
18. Exhaustion T1	2.54	0.91	0.09 **	0.10 **	−0.03	−0.06 **	−0.06 **	0.04	−0.09 **	−0.06 **	−0.22 **	−0.20 **	−0.19 **	0.34 **	0.27 **	0.24 **	0.06 **	0.07 **	0.08 **	0.76		
19. Exhaustion T2	2.52	0.90	0.11 **	0.08 **	−0.03	−0.09 **	−0.07 **	0.03	−0.12 **	−0.06 *	−0.23 **	−0.26 **	−0.21 **	0.28 **	0.35 **	0.27 **	0.08 **	0.08 **	0.08 **	0.58 **	0.78	
20. Exhaustion T3	2.57	0.91	0.13 **	0.13 **	−0.04	−0.08 **	−0.10 **	−0.00	−0.08 **	−0.06 **	−0.22 **	−0.23 **	−0.24 **	0.28 **	0.29 **	0.35 **	0.06 **	0.05 *	0.08 **	0.54 **	0.63 **	0.79

^a^ Control variables are shown at T1 only. Gender (0 = male, 1 = female), education level (0 = low or medium, i.e., school education or vocational training, 1 = high, i.e., academic degree or master craftsman’s diploma), living with a partner (0 = no, 1 = yes), underage child in household (0 = no, 1 = yes), regular day work (0 = no, i.e., working hours usually not between 7 am and 7 pm, 1 = yes, i.e., working hours usually between 7 am and 7 pm), requirement level according to the German classification of occupations (0 = unskilled or semi-skilled activities or specialist activities, 1 = complex specialist activities or highly complex activities). ^b^ Cronbach’s α of the scales are shown on the diagonal. * *p* < 0.01. ** *p* < 0.001.

**Table 2:** Fit indexes and model comparisons for WTC, IWHI/IHWI, and exhaustion (*n* = 3341–3390).

	IWHI						IHWI					
	chi^2^	df	RMSEA	CFI	BIC	Δchi^2^(df) ^a^	chi^2^	df	RMSEA	CFI	BIC	Δchi^2^(df) ^a^
M0: Stability model	392.412	21	0.072	0.965	73,791		309.949	21	0.064	0.968	69,822	
M1: Causal mediation	323.270	19	0.069	0.971	73,731	vs. M0: 71.076(2) **	297.678	19	0.066	0.970	69,827	vs. M0: 10.677(2) *
M2: Reversed mediation	275.906	17	0.067	0.975	73,696	vs. M1: 48.189(2) **	271.718	17	0.066	0.972	69,817	vs. M1: 25.163(2) **
M3: Direct effect	225.107	16	0.062	0.980	73,647	vs. M2: 54.232(1) **	216.748	16	0.061	0.978	69,763	vs. M2: 60.563(1) **
M4: Reversed direct effect	219.242	15	0.063	0.980	73,651	vs. M3: 4.595(1) n.s.	208.534	15	0.062	0.979	69,764	vs. M3: 7.239(1) *
M5: Final model	512.043	122	0.031	0.971	71,819		504.774	113	0.032	0.967	68,121	

^a^ Chi-square difference calculated with the Satorra-Bentler chi-square difference test. * *p* < 0.01. ** *p* < 0.001. df = degrees of freedom; RMSEA = root-mean-square error of approximation; CFI = comparative fit index; BIC = Bayesian information criterion.

**Figure A1:**
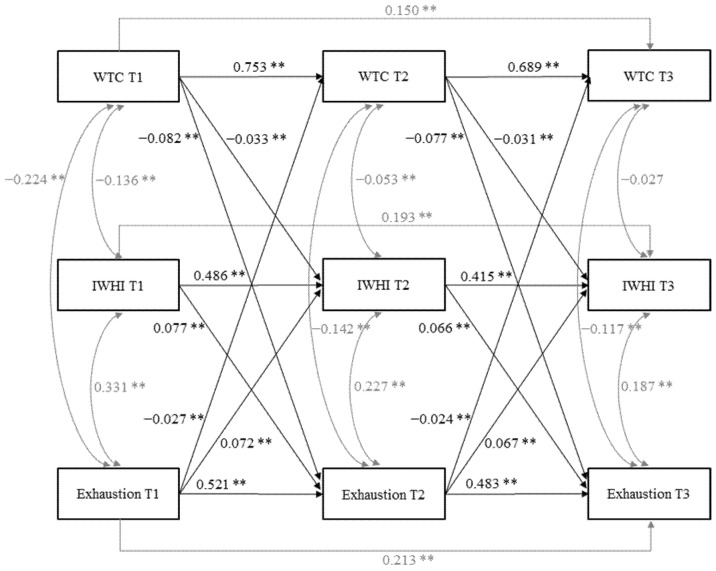
Final model (M5) with standardized coefficients for WTC, IWHI, and exhaustion (unbalanced panel). For clarity, control variables (gender, age, education level, living with a partner, underage child in household, weekly working hours, regular day work, and requirement level) are not shown. Control variables were regressed on the constructs at T1 only. * *p* < 0.01. ** *p* < 0.001.

**Figure A2:**
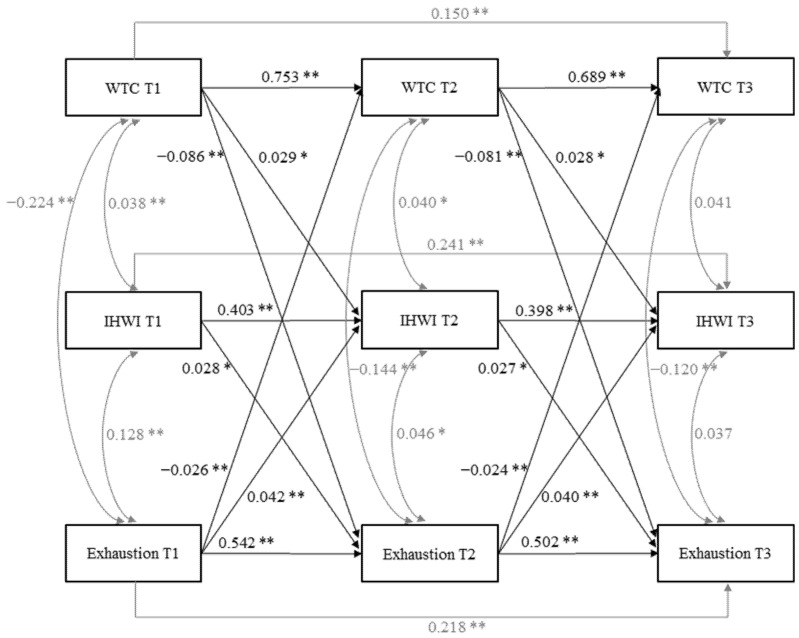
Final model (M5) with standardized coefficients for WTC, IHWI, and exhaustion (unbalanced panel). For clarity, control variables (gender, age, education level, living with a partner, underage child in household, weekly working hours, regular day work, and requirement level) are not shown. Control variables were regressed on the constructs at T1 only. * *p* < 0.01. ** *p* < 0.001.

## Data Availability

The data will be available as a scientific use file (SUF) in 2023 (https://www.baua.de/DE/Angebote/Forschungsdaten/Arbeitszeitbefragung.html).

## Appendix A. Results Based on the Unbalanced Panel

### Appendix A.1. Internal Work-to-Home Interference as a Mediator

Model fit was improved by adding causal mediation pathways via IWHI (M1: WTC→IWHI β = −0.028, *p* < 0.01; IWHI→exhaustion β = 0.070, *p* < 0.001) to the null model (M0; Table A1 shows model fit indices and chi² difference tests). Model fit was further improved by entering reversed mediation pathways (M2: exhaustion→IWHI β = 0.081, *p* < 0.001; IWHI→WTC β = −0.024, *p* < 0.001) and direct effects (M3: WTC→exhaustion β = −0.068, *p* < 0.001). Fit indexes varied in the model with reversed direct effects (M4: exhaustion→WTC β = −0.024, *p* < 0.01), while the chi² statistics indicated a significantly better fit. Hence, the final model (M5) included causal and reversed mediation paths as well as direct and reversed direct effect paths. However, the IWHI-to-WTC parts of the reversed mediation paths were nonsignificant before and after we included control variables and therefore were pruned. Including control variables regressed only on the constructs at T1 had no substantial effect on the estimates; paths of causal mediation remained significant (WTC→IWHI β = −0.030, *p* < 0.001, IWHI→exhaustion β = 0.062, *p* < 0.001; Figure A1). The indirect effect of WTC on exhaustion via IWHI was estimated at −0.002 (SE 0.001, 95% CI −0.003–−0.001, *p* < 0.01; standardized −0.002, SE 0.001).

### Appendix A.2. Internal Home-to-Work Interference as Mediator

Model fit was improved by including causal mediation pathways via IHWI (M1: WTC→IHWI β = 0.017, *p* = 0.012; IHWI→exhaustion β = 0.026, *p* < 0.01) to the null model (M0; see chi² difference test in Table A1, but fit indices results differed). Model fit was further improved by adding reversed mediation pathways (M2: exhaustion→IHWI β = 0.039, *p* < 0.001; IHWI→WTC β = 0.017, *p* = 0.054), direct effects (M3: WTC→exhaustion β = −0.072, *p* < 0.001), and reversed direct effects (M4: exhaustion→WTC β = −0.033, *p* < 0.001). Thus, our final model (M5) included causal and reversed mediation paths as well as direct and reversed direct effect paths. However, we pruned paths from IHWI to WTC because they were nonsignificant before and after we included control variables. Control variables regressed only on the constructs at T1 affected estimates only slightly. Causal mediation paths from WTC to exhaustion via IHWI remained significant (WTC→IHWI β = 0.022, *p* < 0.01, IHWI→exhaustion β = 0.030, *p* < 0.01; Figure A2). The indirect effect was 0.001 (SE 0.000, 95% CI 0.000–0.001, *p* = 0.022; standardized −0.001, SE 0.000).

**Table A1:** Fit indexes and model comparisons for WTC, IWHI/IHWI, and exhaustion (unbalanced panel; *n* = 17,596–17,918).

	IWHI						IHWI					
	chi^2^	df	RMSEA	CFI	BIC	Δchi^2^(df)^a^	chi^2^	df	RMSEA	CFI	BIC	Δchi^2^(df) ^a^
M0: Stability model	485.952	21	0.035	0.968	223,004		355.714	21	0.030	0.974	211,300	
M1: Causal mediation	385.928	19	0.033	0.975	222,912	vs. M0: 102.673(2) **	340.678	19	0.031	0.975	211,305	vs. M0: 13.726(2) *
M2: Reversed mediation	315.256	17	0.031	0.980	222,853	vs. M1: 72.300(2) **	314.047	17	0.031	0.977	211,299	vs. M1: 25.341(2) **
M3: Direct effect	218.061	16	0.027	0.986	222,753	vs. M2: 104.146(1) **	210.550	16	0.026	0.985	211,188	vs. M2: 115.605(1) **
M4: Reversed direct effect	208.591	15	0.027	0.987	222,754	vs. M3: 8.888(1) *	193.208	15	0.026	0.986	211,180	vs. M3: 18.078(1) **
M5: Final model	506.305	72	0.019	0.981	215,495		612.906	70	0.021	0.972	204,481	

^a^ Chi-square difference calculated with the Satorra-Bentler chi-square difference test. * *p* < 0.01. ** *p* < 0.001. df = degrees of freedom; RMSEA = root-mean-square error of approximation; CFI = comparative fit index; BIC = Bayesian information criterion.

**Table A2:** Results from multiple logistic regression analysis with study variables and control variables (both at T1) on dropout (stayer = 0, leaver = 1; *n* = 17,256). R² = 0.020 (Cox & Snell), 0.032 (Nagelkerke). Model χ^2^(12) = 346.730 **.

Variable	B(SE)	exp b	95% CI
Constant	2.478 (0.165) **	11.913	
Gender	0.072 (0.044)	1.075	0.987–1.171
Age	−0.019 (0.002) **	0.981	0.977–0.985
Education level	−0.323 (0.048) **	0.724	0.658–0.796
Living with partner	0.024 (0.047)	1.024	0.935–1.123
Child in household	−0.328 (0.044) **	0.720	0.661–0.785
Weekly working hours	0.002 (0.002)	1.002	0.998–1.006
Regular day work	0.054 (0.057)	1.055	0.944–1.179
Requirement level	−0.272 (0.050) **	0.762	0.690–0.841
WTC	−0.015 (0.019)	0.985	0.949–1.023
IWHI	0.002 (0.019)	1.002	0.965–1.041
IHWI	0.047 (0.025)	1.049	0.997–1.102
Exhaustion	0.019 (0.024)	1.019	0.973–1.067

* *p* < 0.01. ** *p* < 0.001.

**Table A3:** Results from Mann-Whitney U tests and Fisher’s exact tests comparing means of study variables and control variables (both at T1) for stayers and leavers (*n* for stayers = 3354–3390; *n* for leavers = 14,283–14,531).

Variable ^a^	M (Stayers)	SD (Stayers)	M (Leavers)	SD (Leavers)	z	*p*
Gender	0.48	0.50	0.50	0.50		0.007
Age	47.35	8.60	45.58	11.17	−6.46	0.000
Education level	0.58	0.49	0.45	0.50		0.000
Living with partner	0.74	0.44	0.71	0.46		0.000
Child in household	0.39	0.49	0.34	0.47		0.000
Weekly working hours	39.09	10.49	38.67	11.38	−2.09	0.037
Regular day work	0.85	0.36	0.83	0.38		0.006
Requirement level	0.56	0.50	0.44	0.50		0.000
WTC	3.40	1.10	3.31	1.09	−4.44	0.000
IWHI	2.53	1.10	2.49	1.12	−2.06	0.040
IHWI	1.85	0.76	1.91	0.84	−2.34	0.020
Exhaustion	2.54	0.91	2.57	0.93	−1.47	0.142

^a^ Gender (0 = male, 1 = female), education level (0 = low or medium, i.e., school education or vocational training, 1 = high, i.e., academic degree or master craftsman’s diploma), living with a partner (0 = no, 1 = yes), underage child in household (0 = no, 1 = yes), regular day work (0 = no, i.e., working hours usually not between 7 am and 7 pm, 1 = yes, i.e., working hours usually between 7 am and 7 pm), requirement level according to the German classification of occupations (0 = unskilled or semi-skilled activities or specialist activities, 1 = complex specialist activities or highly complex activities).
